# Immune responses in COVID-19 and tuberculosis coinfection: A scoping review

**DOI:** 10.3389/fimmu.2022.992743

**Published:** 2022-08-26

**Authors:** Kevin Flores-Lovon, Brando Ortiz-Saavedra, Luis A. Cueva-Chicaña, Shalom Aperrigue-Lira, Elizbet S. Montes-Madariaga, David R. Soriano-Moreno, Brett Bell, Rodney Macedo

**Affiliations:** ^1^ Universidad Nacional de San Agustín de Arequipa, Arequipa, Peru; ^2^ Grupo de Investigación en Inmunología – GII, UNSA, Arequipa, Peru; ^3^ Unidad de Investigación Clínica y Epidemiológica, Universidad Peruana Unión, Lima, Peru; ^4^ Department of Radiation Oncology, Albert Einstein College of Medicine, Bronx, NY, United States

**Keywords:** COVID-19, SARS-CoV-2, tuberculosis, *Mycobacterium tuberculosis*, coinfection, immunity

## Abstract

**Background and aim:**

Patients with COVID-19 and tuberculosis coinfection are at an increased risk of severe disease and death. We therefore sought to evaluate the current evidence which assessed the immune response in COVID-19 and tuberculosis coinfection

**Methods:**

We searched Pubmed/MEDLINE, EMBASE, Scopus, and Web of Science to identify articles published between 2020 and 2021. We included observational studies evaluating the immune response in patients with tuberculosis and COVID-19 compared to patients with COVID-19 alone.

**Results:**

Four cross-sectional studies (372 participants) were identified. In patients with asymptomatic COVID-19 and latent tuberculosis (LTBI), increased cytokines, chemokines, growth factors and humoral responses were found. In addition, patients with symptomatic COVID-19 and LTBI had higher leukocytes counts and less inflammation. Regarding patients with COVID-19 and active tuberculosis (aTB), they exhibited decreased total lymphocyte counts, CD4 T cells specific against SARS-CoV-2 and responsiveness to SARS-CoV-2 antigens compared to patients with only COVID-19.

**Conclusion:**

Although the evidence is limited, an apparent positive immunomodulation is observed in patients with COVID-19 and LTBI. On the other hand, patients with COVID-19 and aTB present a dysregulated immune response. Longitudinal studies are needed to confirm these findings and expand knowledge.

## Introduction

**Figure 1:**
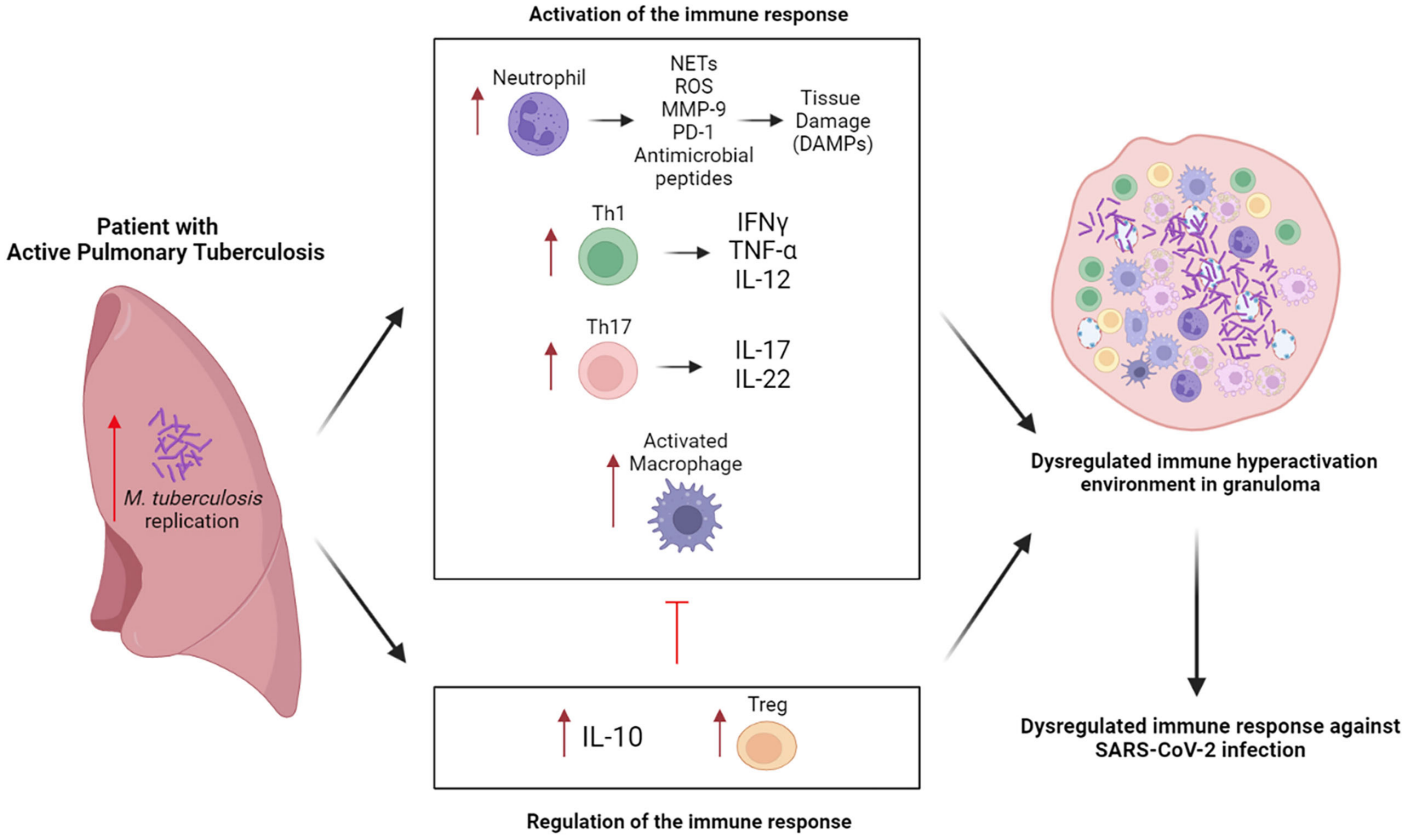
The immune response in COVID-19 and active tuberculosis. The Th1 response potentiates macrophage activity and the Th17 response favors the recruitment and activation of neutrophils with release of harmful products to the host tissue, further exacerbating the inflammatory process. To appease these responses, an elevated production of IL-10 and differentiation of Treg occurs, however, this generates a low immunologic potential. This response caused by M. tuberculosis generates an unfavorable immune microenvironment for theresnponse against SARS-CoV-2. Created with BioRender.com.

According to the World Health Organization, nine million people were infected with tuberculosis worldwide during the year 2020 (1). In addition, approximately 1% of COVID-19 cases present active tuberculosis (aTB) (2), this being a risk factor for developing severe disease and death in patients with COVID-19 (2, 3). Patients with pulmonary aTB present heterogeneous granulomatous lesions, where there is a vigorous inflammatory process characterized by cell-mediated immunity, with production of key cytokines, including TNF-α, IFN-γ and IL-12, to fight *Mycobacterium tuberculosis* (Mtb) infection together with increased activity of immunoregulatory mechanisms (**Figure 1**) (4–6). This chaotic environment coupled with the COVID-19 coinfection could cause further damage to lung tissue. Similarly, in patients with latent tuberculosis (LTBI), T cells and macrophages produce IFN-γ, TNF-α and IL-2 that are responsible for infection control by granuloma formation to contain the pathogen (7, 8). In these patients, SARS-CoV-2 infection probably affects immune regulation in the granuloma. Mild SARS-CoV-2 infection produces an immune response characterized by increased T cell counts with increased functional capacity, along with an increased dendritic cell count and decreased natural killer (NK) cell count (9). Although, in severe SARS-CoV-2 infection there is an initial hypofunctional response which is unable to control the infection followed by a late hyperfunctional response characterized by a cytokine storm (9, 10). This unique immune response to SARS-CoV2 infection has different consequences in the significant proportion of patients globally who experience Mtb. Therefore, it is necessary to better understand the immune response triggered during COVID-19 and tuberculosis coinfection. However, there are few studies evaluating the immunopathology in this context. Hence, we conducted this scoping review to synthesize the current evidence which evaluated the immune response in COVID-19 and tuberculosis coinfection.

## Methods

We conducted the present scoping review following the recommendations of the Preferred Reporting Items for Systematic and Meta-Analysis extension for Scoping Reviews (PRISMA-ScR) (11), and the methodological guidelines of the Joana Briggs Institute (12). The protocol was registered in the Figshare platform (https://n9.cl/unzch). The PRISMA-ScR Checklist is found in **Supplementary Material 1**.

### Inclusion and exclusion criteria

We included observational studies (cohort, cross-sectional, case-control) in patients older than 18 years with diagnosis of COVID-19 by serology or positive RT-PCR for SARS-CoV-2. For aTB diagnosis we considered a positive sputum test, culture of respiratory specimens, molecular test (GeneXpert/GenoType) or specific histological findings. For LTBI diagnosis we considered a QuantiFERON test or positive Mantoux test. Studies without a control group considering patients with COVID-19 were excluded.

### Search strategy

We conducted the literature search in Pubmed/MEDLINE, EMBASE, Scopus and Web of Science databases to identify studies published during the period from January 1st, 2020 to December 10th, 2021. We did not apply language restriction. Search terms were grouped into two categories, “Tuberculosis” and “COVID-19”. The complete strategy for each database is available in **Supplementary Material 2**.

### Selection of studies

Articles found in the systematic search were imported into Zotero software where duplicates were removed. Rayyan software was used for article selection. Two authors (BOS, LAQC) independently reviewed titles and abstracts to identify relevant studies for inclusion. Included studies were full text reviewed independently by two authors (ESMM, SAL) and disagreements were resolved by consensus with a third author (DRSM). In addition, we screened references of included studies to identify potentially eligible studies.

### Data extraction

We created a data extraction sheet in Microsoft Excel and significant data were extracted independently by two authors (KFL, BOS). Data extracted included author, year of publication, country, study design, sample size, diagnostic criteria for tuberculosis and COVID-19, COVID-19 severity, tuberculosis type, sample characteristics (age, sex), and outcomes of interest. The results are summarized in narrative form and in tables.

## Results

### Study characteristics

**Figure 2:**
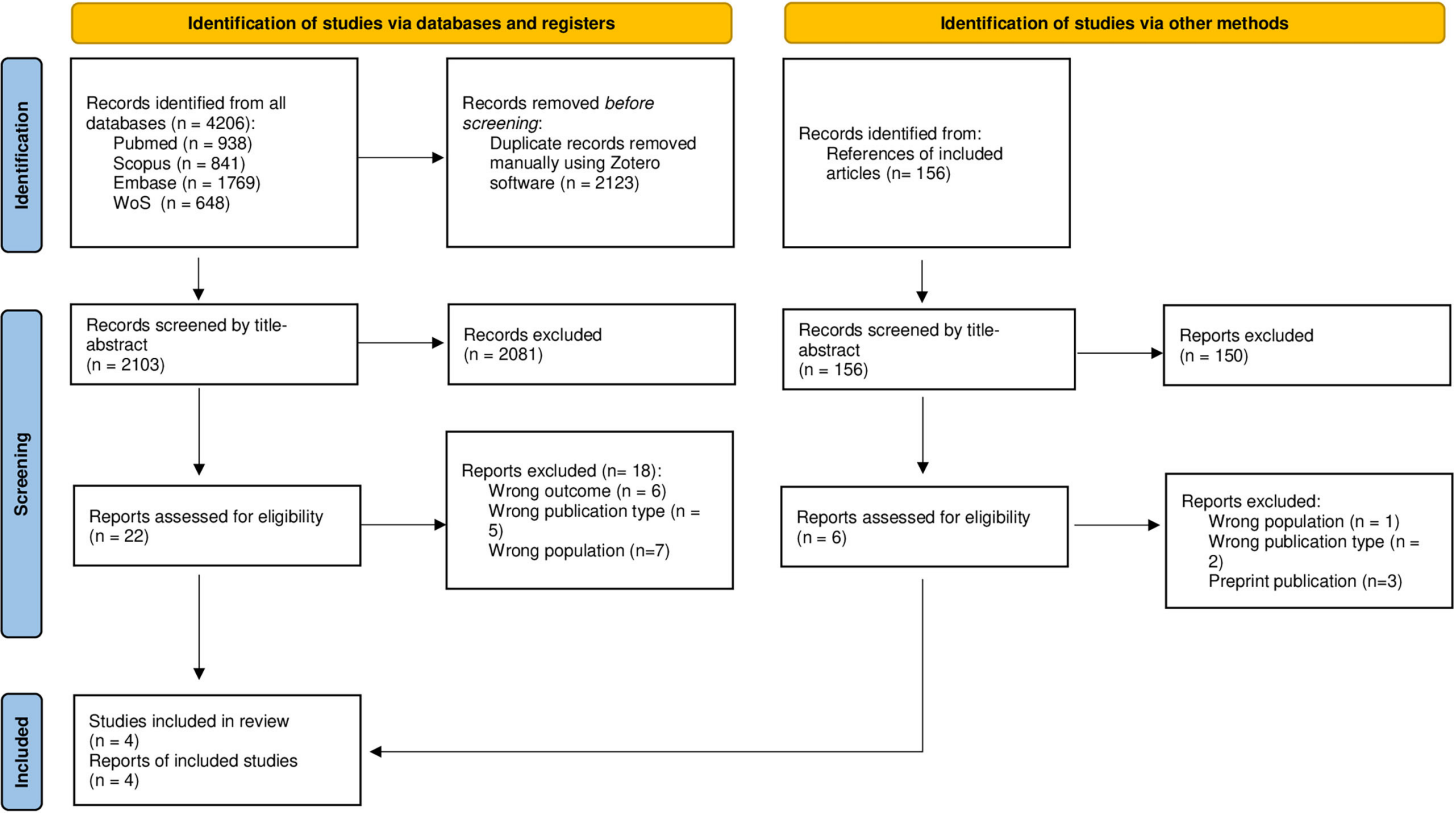
Flow diagram summarizing the process of literature search and selection.

Initially, we reviewed 2103 studies by title and abstract, of which 22 were chosen for full-text review, the reasons for exclusion are found in **Supplementary Material 3**. Finally, we included four cross-sectional studies in the present review (13–16). The selection process is summarized in **Figure 2**. The number of participants ranged from 60 to 133 with a total of 372 to predominantly male, and the mean ages ranged from 43 to 68 years, these characteristics are found in **Table 1**. Two studies evaluated patients with LTBI (13, 14), one patient with aTB (15) and other patients with LTBI and aTB (16). One study evaluated asymptomatic COVID-19 patients (13), and the other three evaluated mild to severe symptomatic patients (14–16). Among the outcomes included were serum levels of cytokines, chemokines, growth factors, laboratory inflammatory markers, humoral response, antigen responsiveness and lymphocytes (**Table 2**).

**Table 1:** Characteristics of the studies that evaluated the immune response in tuberculosis and COVID19 coinfection (n=4).

Study-year	Country	Study design	Tuberculosis diagnosis	COVID-19 diagnosis	COVID-19 severity	Sample size	Groups	Sex male (%)	Age (Mean ± SD) years
Rajamanickam (13) 2021	India	Cross-sectional	LTBI: QuantiFERON TB Gold in tube	IgG +	Asymptomatic	133	COVID19/LTBI + (n=61)	49.2	68 ± 12.9
							COVID19/LTBI - (n=72)	62.5	67 ± 12.9
Petrone (16) 2021	Italy	Cross-sectional	aTB: Sputum culture, molecular test, histopathological findings, clinical and radiological criteria.	Nasopharyngeal swab	Mild, Moderate, Severe and Critical	84	COVID19/aTB + (n=10)	60.0	43 ± 12.9
							COVID19/LTBI - (n=11)	36.4	60 ± 14.4
			LTBI: QuantiFERON Plus or radiological				COVID19 (n=63)	66.7	54 ± 14.4
Riou (15) 2021	South Africa	Cross-sectional	Not specified	RT-PCR test and serology		133	COVID19/aTB + (n=15)	57.9	51 ± 10.5
					Mild, moderate, severe				
							COVID19/aTB - (n=80)		

Madan (14) 2021	India	Cross-sectional	LTBI: Mantoux tuberculosis skin test	Not specified	Mild and severe	60	COVID19/LTBI + (n=15)	83.3	46 ± 15.2
							COVID19/LTBI - (n=45)		

LTBI, Latent Tuberculosis.

aTB, Active tuberculosis.

**Table 2:** Outcomes evaluated in studies assessing immune response in tuberculosis and COVID-19 co-infection (n=4).

Study	Immune response parameters evaluated		Findings with significant difference (p<0.05).
Rajamanickam et al. (13)	Cytokines	IFN-γ, IL-2, TNF-α, IL-1α, IL-1β, IFN-α, IFN-β, IL-3, IL-4, IL-5, IL-6, IL-7, IL-10, IL-12, IL-13, IL-15, IL-17, IL-25 y IL-33, G-SCF, GM-CSF, and IL-1Rα	The levels of IFN-γ, IL-2, TNF-α, IL-1α, IL-1β, IFN-α, IFN-β, IL-6, IL-12, IL-15, IL-17, IL-3, GM-CSF, IL-10, IL-25 and IL-33 were higher in LTBI/COVID19 *vs.* COVID19.
	Chemokines	CCL2, CCL3, CCL4, CCL5, CCL11, CCL19, CCL20, CXCL1, CXCL2, CXCL8, CXCL10, and CX3CL1.	CCL3 and CXCL10 levels were higher in LTBI/COVID-19 *vs.* COVID-19.
	Growth factors	VEGF, EGF, FGF-2, PDGF-AA, PDGF-BB, TGFα, Fit-3L, GZB, PDL-1, TRAIL, and CD40L	VEGF and TGFα levels were higher in LTBI/COVID-19 *vs.* COVID-19.
	Laboratory inflammatory markers	CRP, α-2- microglobulin, haptoglobin and serum amyloid.	CRP and α-2- microglobulin levels were higher in LTBI/COVID-19 *vs.* COVID-19.
	Humoral response	Ig M, Ig G, Ig A, and neutralization capacity.	Levels of neutralizing antibodies, Ig M, Ig G and Ig A specific against SARS-CoV-2, were higher in LTBI/COVID-19 *vs.* COVID-19.
Petrone et al. (16)	Antigen responsiveness	IFN-γ response to TB1, TB2, CD4S, MIT antigens.	For TB1, IFN-γ level was higher in aTB/COVID-19 and LTBI/COVID-19 *vs.* COVID-19.
			For TB2, IFN-γ level was higher in aTB/COVID-19 and LTBI/COVID-19 *vs.* COVID-19.
			For CD4S, the IFN-γ level was lower in aTB/COVID-19 *vs.* LTBI/COVID-19 and COVID-19.
Riou et al. (15)	Lymphocytes	TCD4/SARS-CoV-2	TCD4/SARS-CoV-2 show lower polyfunctional capacity (INF-γ, IL-2 and TNF-α) in aTB/COVID-19 *vs.* COVID-19.
			The overall phenotype of TCD4/SARS-CoV-2 was different in aTB/COVID-19 *vs.* COVID-19.
		TCD4/Mtb	Number of TCD4/Mtb was lower COVID-19 *vs.* Non COVID-19
Madan et al. (14)	Laboratory inflammatory markers	Absolute lymphocyte counts Absolute and percentage count of neutrophils and monocytes, NRL and CRP, D-dimer, fibrinogen and ferritin.	Absolute monocyte count was higher in LTBI/COVID-19 *vs.* COVID-19.
			NRL was lower in LTBI/COVID1-9 *vs.* COVID-19.
			CRP level was lower in LTBI/COVID-19 *vs.* COVID-19
	Lymphocytes	Absolute and percentage lymphocyte counts	Absolute lymphocyte count was higher in LTBI/COVID-19 *vs.* COVID-19.

CRP, C-reactive protein.

NRL, Neutrophil/lymphocyte ratio.

TCD4/SARS-CoV-2, Specific CD4 T-cell response against SARS-CoV-2.

TCD4/Mtb, Specific CD4+ T-cell response against Mycobacterium tuberculosis.

CD4S, Peptide derived from the SARS-CoV-2 Spike protein.

MIT, Mitogenic antigen.

### Cytokines, chemokines and growth factors

Rajamanickam et al. (13), found that patients with asymptomatic COVID-19 and LTBI had higher levels of serum cytokines including IFN-γ, TNF-α, IL-1α, IL-1β, IL-2, IL-6, IL-12, IL-15, IL-17, IFN-α, IFN-β, IL-3, GM-CSF, IL-10, IL-25 and IL-33, chemokines CCL3 and CXCL10, and growth factors VEGF and TGF-α, compared to patients with only COVID-19 However, for cytokines IL-4, IL-5, IL-13 and IL-1Rα, chemokines CCL2, CCL4, CCL5, CCL11, CCL19, CCL20, CXCL1, CXCL2, CXCL8 and CX3CL1, and growth factors EGF, FGF-2, PDGF-AA, PDGF-BB, Flt-3L, GZB, PDL-1, TRAIL and CD40L there were no significant differences.

### Lymphocytes

Two studies evaluated the total lymphocyte count. Madan et al. (14) found a higher count in patients with symptomatic LTBI and COVID-19 compared to patients with only COVID-19. For the evaluation of specific CD4 T lymphocytes and their functionality, Riou et al. (15) evaluated the SARS-CoV-2-specific CD4+ T lymphocyte response and found that patients with only COVID-19 had a higher polyfunctional CD4+ T lymphocyte capacity (IFN-γ, IL-2 and TNF-α) and differences in the overall phenotype of CD4+ T lymphocytes specific against SARS-CoV-2 compared to patients with COVID-19 and aTB. However, both groups had similar percentages of SARS-CoV-2-specific CD4+ T-cells. Likewise, HLA-DR expression and the percentage of CD4+ T lymphocytes specific against Mtb were both similar between patients with COVID-19 and aTB compared to patients without COVID-19. It should be noted that this study had stratified groups according to HIV-positive or negative status, so all results that included HIV+ patients were not considered in the present review.

### Antigen responsiveness

Petrone et al. (16) found that patients with symptomatic COVID-19 and tuberculosis (aTB or LTBI) had significantly higher IFN-γ levels in response to TB1, and TB2 antigens compared to COVID-19-only patients. On the other hand, patients with only COVID-19 or COVID-19 with LTBI coinfection presented significantly higher levels of IFN-γ in response to CD4S antigen compared to patients with COVID-19 and aTB coinfection. No significant differences were found when comparing the response to MIT antigen between the groups.

### Humoral response

Rajamanickam’s study (13) quantitatively evaluated serum levels of IgM, IgG, IgA and neutralizing antibodies specific against SARS-CoV-2, finding that all were elevated in patients with COVID-19 and LTBI compared to patients with only COVID-19.

### Inflammatory laboratory markers

Rajamanickam et al. (13) found that patients with asymptomatic COVID-19 and LTBI had higher C-reactive protein (CRP) and α-2 microglobulin levels compared to patients with COVID-19 alone. However, Madan et al. (14) found that patients with symptomatic COVID-19 and LTBI had lower CRP levels, in addition to higher monocyte values and decreased neutrophil/lymphocyte ratio (NLR) compared to the COVID-19 alone group. Also, D-dimer and ferritin values were decreased, but this difference was not significant. Total leukocytes, absolute neutrophils and platelets were similar between groups (14).

## Discussion

This review identifies and characterizes the cellular and humoral immune response observed in COVID-19 and tuberculosis coinfection. Based on four cross-sectional studies, patients with asymptomatic COVID-19 and LTBI exhibited a more prominent immune response against SARS-CoV-2, characterized by increased serum cytokines, chemokines, growth factors and immunoglobulins when compared with patients with only COVID-19 (13). Additionally, patients with symptomatic COVID-19 and LTBI had higher leukocytes counts in comparison to only COVID-19 patients (14). In contrast, COVID-19 and aTB coinfection, exhibited lower polyfunctional capacity of CD4+ T lymphocytes with less responsive to SARS-CoV-2 specific antigens in comparison with patients with only COVID-19 (15, 16).

### Cytokines and chemokines

At the onset of SARS-CoV-2 infection, the innate immune system produces cytokines and chemokines to inhibit viral replication. This initial response is characterized by elevated serum levels of IFN-α, TNF, and IFN-γ which is typically more prominent and effective in patients who ultimately survive than in patients who die of COVID-19 (17, 18). In the present review, the study by Rajamanickam et al. (13) studied patients with asymptomatic COVID-19 and found that patients with LTBI and COVID-19 coinfection had higher production of protective (IL-1α, IL-1β, IL-2, IL-12, IL-17, TNF-α, IFN-α, IFN-β, IFN-γ) and regulatory (IL-4, IL-10, IL-25, IL-33) cytokines than patients with COVID-19 alone. This increased cytokine production coincides with the increased cytokine production in LTBI as part of the response against Mtb (16), independent of SARS-CoV-2 infection. This study also found that LTBI/COVID-19 versus COVID-19 patients had higher levels of CCL3 and CXCL10 chemokines, which are critical for T cell migration and Th1 immune response. Furthermore, it should be noted that we have not found studies evaluating cytokine and chemokine production in patients with COVID-19 and aTB coinfection, where their production might be different. Differences were particularly apparent in patients with severe COVID-19, where cytokine storm is known to trigger unfavorable outcomes such as acute respiratory distress syndrome (ARDS), sepsis and coagulopathy (9). Importantly, IL-2 and IL-12 levels are significantly higher in patients with asymptomatic and mild COVID-19 versus moderate and severe disease, while IL-6 levels are correlated with increased severity and poor outcomes in COVID-19 (19, 20). These results, suggest that despite the increase of IL-6 seen in LTBI and COVID-19 coinfection that could worsen the disease, these patients also increase IL-2, IL-12 and other cytokines which will balance the negative IL-6 effects.

### Lymphocytes

An adequate immune response at the onset of SARS-CoV-2 infection leads to T-lymphocyte elevation (17). However, in patients with severe COVID-19, lymphocyte counts decrease and are associated with a worse prognosis with respect to disease severity (21). The study by Madan et al. (14) found that patients with symptomatic COVID-19 and LTBI had higher absolute lymphocyte counts compared to patients with COVID-19 alone. In patients with LTBI, prior exposure to and control of Mtb produces trained innate immunity leading to early development of adaptive immunity (22), along with the development of heterologous immunity with cross-reactivity and activation of memory T lymphocytes by the presence of SARS-CoV-2 peptides (23, 24). This early response would avoid the intense proliferation, migration, apoptosis and late extravasation of lymphocytes, these mechanisms cause the characteristic lymphopenia in severe COVID-19 disease (17), which is associated with higher mortality (25).

However, with COVID-19 and aTB infection, the cellular response seems different. Petrone et al. and Riou et al. report that patients with COVID-19 and aTB had decreased absolute lymphocyte counts (16), CD4+ T lymphocytes specific against SARS-CoV-2 (14) and polyfunctional CD4+ T lymphocytes (IFN-γ+, IL-2+, TNF-α+) (15), compared to patients with COVID-19 alone. This group of patients mostly presented with moderate COVID-19 disease, which explains a marked decrease in the percentage of polyfunctional T lymphocytes (IFN-γ+, IL-2+, TNF-α+) (26). Likewise, it has been reported that patients with aTB have a low total lymphocyte count (27) and decreased Mtb-specific polyfunctional T lymphocyte (IFN-γ+, IL-2+, TNF-α+) capacity (28), with hyperactivation of regulatory T lymphocytes. This suggests that in COVID-19 and aTB there is a distorted hyperinflammatory synergistic state with low immune potential that would alter the polyfunctional capacity and development of CD4-specific T lymphocytes specific against SARS-CoV-2.

It has previously been hypothesized that SARS-CoV-2 infection could reactivate LTBI (29). The study by Riou et al. (15) found that patients with COVID-19 had lower numbers of CD4 T lymphocytes specific against Mtb compared with patients without COVID-19, which could mean that cellular defense against Mtb is decreased with SARS-CoV-2 coinfection. However, among the groups of patients with SARS-CoV-2 infection, no significant differences were found in the expression of markers of Mtb-specific CD4 T lymphocyte activation and proliferation (HLA-DR, CD38, and Ki67) (15, 30). Thus, the current evidence is inconsistent and future studies should corroborate these findings.

### Responsiveness to SARS-CoV-2 and Mtb antigens

T lymphocytes are the main producers of IFN-γ and their synthesis is stimulated after the specific recognition of antigens from prior exposures (31). The study by Petrone et al. (16) found that in patients with symptomatic COVID-19 and aTB the level of IFN-γ produced in response to SARS-CoV-2 specific CD4S antigen was lower compared to patients with only COVID-19 or COVID-19 and LTBI coinfection. Indeed, this decrease in IFN-γ production after antigen stimulation perfectly correlates with the lower number of polyfunctional CD4+ T cells (IFN-γ+, IL-2+, TNF-α+) that react against CD4S antigens observed by Riou et al. in COVID-19 and aTB coinfection versus only COVID-19 (15). Interestingly, Petrone et al. also showed non-significant higher levels of IFN-γ secreted by CD4S-specific T cells from patients with COVID-19 and LTBI when compared with only COVID-19 group (16). In fact, CD4S and TB1/TB2-specific T cell clones could be the main source of the higher serum levels of IFN-γ observed in patients with COVID-19 and LTBI versus COVID-19 in Rajamanickam et al. (11). Together, these results suggest that a previous exposure to TB as observed in LTBI could be helping T cells responsiveness against SARS-CoV-2.

### Humoral response

T-lymphocyte stimulation plays a key role in B-lymphocyte activation and differentiation, resulting in the production of neutralizing antibodies and affinity maturation (31, 32). Rajamanickam et al. (13) report that patients with asymptomatic COVID-19 and LTBI had higher levels of neutralizing antibodies, IgM, IgG and IgA specific to SARS-CoV-2, compared to the COVID-19 alone group. Similar results were observed in persons immunized against SARS-CoV-2 and BCG (31, 33). This robust humoral response could be due to early recognition of SARS-CoV-2 peptides, together with activation and differentiation of memory T lymphocytes, products of heterologous immunity after previous exposure to Mtb antigens.

### Hematologic and inflammatory laboratory biomarkers

Hematologic and inflammatory biomarkers are useful as predictors of severe disease and mortality due to COVID-19. These include increased leukocytes, decreased lymphocytes and platelets, elevated neutrophil/lymphocyte ratio, and elevated IL-6, CRP, D-dimer, and ferritin (21, 34) Rajamanickam et al. (13) evaluated patients with asymptomatic COVID-19 and found that patients with LTBI had elevated CRP and α-2 microglobulin levels compared to patients to patients with only COVID-19. On the other hand, Madan et al. (14) found that patients with symptomatic COVID-19 and LTBI had increased neutrophil/lymphocyte ratio and monocyte counts, with decreased CRP, compared to patients with COVID-19 alone. A possible explanation for these results is that in the study the patients are not in the acute phase of the infection (9), in addition, in this same group an elevation of IL-6 was found, which would elevate hepatic synthesis of CRP (35). Meanwhile, in the study by Madan et al. (14) patients with symptomatic LTBI and COVID-19, although they are in an acute stage of infection, the time of infection is not specified, so the values could correspond to the second stage of the immune response against SARS-CoV-2 (9).

### Limitations of included studies

The studies included in this review have certain limitations. First, all were cross-sectional, so the lack of longitudinal studies limits the assessment of causality. In addition, the sample sizes were small which limits statistical power. Not all the studies presented the same populations, type of tuberculosis, measurements or the same objectives, so that for most of the outcomes evaluated, consistency between the results could not be verified. In addition, none of the studies performed analyses adjusted for confounding factors.

### Implications and recommendations

In the present review, we included studies that evaluated the immune response in patients with COVID-19 and tuberculosis compared to patients with only COVID-19. We found that patients with COVID-19 and LTBI develop an immune response that could positively impact the outcome. However, patients with COVID-19 and aTB exhibited a reduced immune response, characterized by lower IFN-γ response to SARS-CoV-2 specific antigens and lower functional response of lymphocytes that will lead to higher morbidity and mortality. Based on these findings, we propose intervention strategies to identify therapeutic targets and immune-monitoring of T cell specific response against TB and COVID-19 antigens to reduce mortality in patients with coinfection. In addition, we recommend longitudinal studies in other populations, to separately analyze patients with LTBI and aTB and at different stages of severity by COVID-19 to better understand the pathophysiology. Further studies are also needed to understand how COVID-19 might reactivate LTBI or exacerbate aTB. Also, there is a lack of evidence for most of the outcomes evaluated. In patients with asymptomatic COVID-19 and LTBI there is an absence of data on cell counts and antigen responsiveness. In patients with symptomatic COVID-19 and LTBI there is an absence of data on cytokines, humoral response, and antigen responsiveness. Finally, in patients with COVID-19 and aTB no information was found on cytokines, humoral response, and cell counts.

### Limitations and strengths

Among the limitations presented by this scoping review, we have the small number of eligible studies according to the selection criteria and with such a small sample, there is high heterogeneity in the composition of the groups and in the used parameters to evaluate the immune response. In addition, we did not evaluate articles in the gray literature. Regarding strengths, the present study was conducted according to the JBI and PRISMA-ScR guidelines, which ensure methodological soundness and quality of the results. Also, all review processes were performed in duplicate.

## Conclusions

The immune response in COVID-19 and tuberculosis coinfection is complex and is influenced by both innate and adaptive immunity. The results of the current evidence indicate that in LTBI patients, positive immunomodulation against COVID-19 occurs, probably by trained innate immunity and crossed heterologous immunity. On the other hand, patients with aTB would have a lower SARS-CoV-2 specific response and lower lymphocyte function which would not effectively control the infection. There are still several gaps in knowledge and further longitudinal studies are needed to confirm these findings.

## Data availability statement

The original contributions presented in the study are included in the article/**Supplementary Material**. Further inquiries can be directed to the corresponding author.

## Author contributions

KF-L, BO-S, LC-C, SA-L, EM-M, and DS-M conceived and wrote the manuscript. RM and BB contributed to the modification and revision of the manuscript. All authors contributed to the article and approved the submitted version.

## Funding

This work is supported by the US National Institute of Health (NIH) grant R01CA257509 (to BB and RM).

## Acknowledgement

Special thanks to Moises Huarhua, Universidad Peruana Unión, who assisted in reviewing the draft of this article.

## Conflict of interest

The authors declare that the research was conducted in the absence of any commercial or financial relationships that could be construed as a potential conflict of interest.

## Publisher’s note

All claims expressed in this article are solely those of the authors and do not necessarily represent those of their affiliated organizations, or those of the publisher, the editors and the reviewers. Any product that may be evaluated in this article, or claim that may be made by its manufacturer, is not guaranteed or endorsed by the publisher.
